# Biomarkers of Inflammation and Redox Imbalance in Umbilical Cord in Pregnancies with and without Preeclampsia and Consequent Perinatal Outcomes

**DOI:** 10.1155/2021/9970627

**Published:** 2021-11-09

**Authors:** Marilene Brandão Tenório Fragoso, Raphaela Costa Ferreira, Micaely Cristina dos Santos Tenório, Fabiana Andréa Moura, Orlando Roberto Pimentel de Araújo, Nassib Bezerra Bueno, Marília Oliveira Fonseca Goulart, Alane Cabral Menezes de Oliveira

**Affiliations:** ^1^Instituto de Química e Biotecnologia (IQB/UFAL), Rede Nordeste de Biotecnologia (RENORBIO), Universidade Federal de Alagoas, Campus A. C. Simões, BR 104 Norte, Km. 96.7, Tabuleiro dos Martins, CEP 57.072-970 Maceió, Alagoas, Brazil; ^2^Programa de Pós-graduação em Ciências da Saúde, ICBS, Universidade Federal de Alagoas, Campus A. C. Simões, BR 104 Norte, Km. 96.7, Tabuleiro dos Martins, CEP 57.072-970 Maceió, Alagoas, Brazil; ^3^Faculdade de Nutrição, Universidade Federal de Alagoas, Campus A. C. Simões, BR 104 Norte, Km. 96.7, Tabuleiro dos Martins, CEP 57.072-970 Maceió, Alagoas, Brazil; ^4^Instituto de Química e Biotecnologia (IQB/UFAL), Programa de Pós-graduação Em Química e Biotecnologia, Universidade Federal de Alagoas, Campus A. C. Simões, BR 104 Norte, Km. 96.7, Tabuleiro dos Martins, CEP 57.072-970 Maceió, Alagoas, Brazil

## Abstract

**Objective:**

To compare redox imbalance and inflammation biomarkers in umbilical cords from pregnancies with and without preeclampsia (PE) and to analyse their relationships with perinatal outcomes.

**Methods:**

A controlled cross-sectional study was conducted in Maceió, Alagoas, Brazil, that involved pregnant women with PE and a group of women without the disease, through the application of a standardized questionnaire. After delivery, umbilical cord samples were collected to measure antioxidant defense, products from oxidative damage, and inflammation biomarkers such as myeloperoxidase (MPO), interleukin- (IL-) 6, IL-8, IL-10, and tumor necrosis factor-alpha (TNF-*α*). Statistical analyses were performed using Stata version 13.0 software and IBM Statistical Package for the Social Sciences (SPSS) 20.0, adopting a 95% confidence level (*α* = 0.05), with the chi-square test, the Wilcoxon–Mann–Whitney test, and the multinomial and Poisson regression tests.

**Results:**

One hundred PE pregnant women and 50 women without the disease were studied. The umbilical cords from PE pregnancies showed higher levels of reduced glutathione (GSH) (*p* ≤ 0.001), glutathione peroxidase (GPx) (*p* = 0.016), and malondialdehyde (MDA) (*p* = 0.028) and lower levels of IL-6 (*p* = 0.030) and TNF-*α* (*p* ≤ 0.001) than the other group, with some associations among these biomarkers with perinatal outcomes.

**Conclusion:**

The higher levels of GSH and GPx, in addition to the lower levels of IL-6 and TNF-*α*, found in the PE umbilical cord, may result from adaptive mechanisms to maintain the oxidative and inflammatory balance; however, despite these changes, the damage to the cell membranes was not minimized, as the MDA level was higher in women with PE than in women without the disease. This implies that a redox imbalance is present, confirming that other physiological and adaptive mechanisms are being activated to preserve foetal health. Therefore, the present work unveils an important role of the umbilical cord in controlling redox imbalance and inflammation in PE pregnancies. Our results reinforce the necessity for continuous research on GSH as a protective compound for the perinatal outcome, especially in PE women.

## 1. Introduction

The umbilical cord is a vital structure for foetal development, as it provides the only connection with the placenta. The outer part presents a layer of amniotic epithelium that surrounds a core of mucoid connective tissue, namely, Wharton's jelly (WJ), capable of filling the entire tissue space. WJ does not contain other blood or lymph vessels and is not innervated. As a connective tissue, WJ is suited for producing only mesenchymal cells, which comprise the functional myofibroblasts of the tissue and their precursors. WJ surrounds three vessels, two arteries responsible for transporting nutrients and oxygenated blood to the foetus, and a vein, which conducts deoxygenated blood and waste products back to the placenta. The umbilical vessels are comprised of an intimate tunic and a medium tunic, different from other vessels, which also have an adventitious tunic [1–4].

Scientists have demonstrated that structural and functional changes in the umbilical cord may be associated with pathological conditions. For example, diseases such as gestational diabetes mellitus and preeclampsia (PE), which lead to adverse perinatal outcomes such as intrauterine growth restriction (IUGR) and foetal death, are influenced by the cord length and width, WJ area, type of cord insertion, cord knot, morphometry, and the flow parameters of the umbilical vessels [5–7].

The aetiology of PE is not fully understood, but it is known that a deficient placentation process occurs at the beginning of pregnancy, resulting in inadequate remodeling of the uterine spiral arteries, which compromises the supply of oxygenated blood to the foetoplacental unit. As a consequence, hypoxia/reperfusion occurs in the organ, which leads to high production of reactive oxygen species (ROS), leading to oxidative stress in addition to inflammation and endothelial dysfunction. Such changes culminate in the occurrence of adverse perinatal outcomes, such as IUGR, premature birth, newborns small for gestational age, and other complications, including maternal and foetal deaths [8–12]. It is also important to highlight that redox homeostasis depends on a number of factors, including gender, age, disease, and pregnancy, emphasizing, therefore, that PE is a factor able to interfere in this redox homeostasis [9, 13].

PE is currently considered a public health problem that is associated with high maternal and perinatal morbidity and mortality. Changes occur in the extracellular matrix that lead to increased vascular resistance in the foetoplacental circulation. As a result, changes in umbilical cord morphology and composition occur in PE by reducing the area of the umbilical vein and WJ, with narrower cords evident in early-onset PE [4, 14, 15].

In addition, there is an increase in collagen deposition, a reduction in elastin, a thickening of the vessel walls, and the migration of smooth muscle cells. In the PE umbilical cord artery, a decrease in the levels of collagen-degrading enzymes, such as the matrix metalloproteinases, favours changes in the collagen-elastin relationship. Additionally, there is an increase in the proteoglycan amount associated with the reduction of hyaluronic acid, causing WJ to lose its ability to retain water and resist compression. Therefore, the observed changes in the umbilical cord of pregnant women with PE lead to premature deterioration of these tissues, which can result in or contribute to the haemodynamic changes characteristic of PE, as well as favour ROS passage to the foetus through the umbilical cord [4, 16–18].

Despite not being a frequent target of research related to PE, the umbilical cord constitutes a primary vascular structure, responsible for carrying oxygenated blood and nutrients to the foetoplacental unit, as well as foetal metabolites for excretion. As such, changes in its shape and function reflect complications affecting maternal-foetal health [19]. To date, no articles have been published concerning the role of the umbilical cord in maintaining redox and inflammatory balance in women with PE and its consequences on foetal outcomes, which gives a strong support to the present work. It is necessary to obtain data about the pathophysiology of the disease, the role of oxidative stress and inflammation in the umbilical cord, and possible changes in its function, as a way to further understand its role, aiming at minimizing the damage to foetal health from PE. Therefore, this study is aimed at evaluating the markers of redox imbalance and inflammation in umbilical cords of pregnancies with PE, to compare them with those without the disease and analyse their relationship with perinatal outcomes.

## 2. Materials and Methods

### 2.1. Experimental: Reagents and Equipment

A superoxide dismutase (SOD) assay kit-WST® was purchased from Sigma-Aldrich. Cytokine kits were obtained from PeproTech® (PeproTech Brasil FUNPEC, Ribeirão Preto, SP, BR), protease inhibitor cocktail tablets were obtained from Roche® (Germany), and radioimmunoprecipitation assay (RIPA) buffer was obtained from Cell Signaling Technology®. All other chemicals and enzymes were purchased from Sigma-Aldrich® (St. Louis, USA).

The high-performance liquid chromatography system (HPLC) (LC-20 AT-Prominence, Shimadzu) coupled to a UV detector (Shimadzu, Serial no. L201550) was used. A biofreezer from the VIP Series by Sanyo was used. The spectrofluorometer was manufactured by Thermo Fisher Scientific® (Multiskan), who also supplied a Filizola® digital balance, with a capacity of 150 kg and 100 g accuracy and a stadiometer with a 2 m length and 0.1 cm precision.

### 2.2. Study Design and Ethical Aspects

This was a controlled cross-sectional study carried out in 2017 in the city of Maceió, AL, Brazil. The present work is part of a larger research project financed by the Research Program for the Unified Health System, with approval by the Ethics Committee in Research of the Federal University of Alagoas (process no. 35743614.1.0000.5013). It is important to mention that an article has already been published containing the data referring to the placenta analysis by our research group [12] and that this study used the same PE women, women without the disease, and their newborns [12]. Their umbilical cords are herein analysed for the first time.

With regard to the proportion of the sample size between PE and women without the disease groups, according to Tenny et al. [20] and Munnangi and Boktor [21], there is no standard concerning the number of cases and the comparative group, and this proportion can reach up to 4 : 1. However, groups need to have similar characteristics, such as sex and age, differing only in the disease presence or absence. Therefore, the 1 : 1 ratio is optional, and the 2 : 1 ratio used was considered to be adequate.

### 2.3. Inclusion and Exclusion Criteria

The study group included pregnant women diagnosed with PE, and their identification was carried out through medical records following the criteria of the American College of Gynecology and Obstetrics (ACOG) [22] and adjusted posteriorly according to Brown et al. [23]. Women with HELLP (hemolysis, elevated liver transaminases, and thrombocytopenia) syndrome [24], eclampsia, severe general conditions, smokers, twin pregnancies, or other conditions capable of influencing pregnancy outcomes, such as preexisting or gestational diabetes mellitus, cardiovascular and autoimmune diseases, and infections, were not included in this study. Additionally, healthy pregnant women were included as a comparative group. They were recruited in the same maternity of the PE cases. The presence of gestational or pregestational diseases and smoking habits were the exclusion criteria for the women without the disease group. The majority of them were already close to childbirth. Thus, they were invited to participate in the study, answered the questionnaire, and were monitored until the moment of delivery, where the umbilical cord samples were collected.

### 2.4. Data Collection and Classification

The selection of participants took place by an analysis of their medical records to identify pregnant women diagnosed with PE, according to the inclusion criteria previously defined. Then, properly trained researchers approached these women and invited them to participate in the study. After accepting and signing the informed consent form, a standardized questionnaire was used to gather socioeconomic (maternal age, education, and family income) and obstetric (gestational age, presence of complications in the current pregnancy, and information on previous pregnancies) information, in addition to information about the current maternal nutritional status (height, current weight, and calculation of the body mass index (BMI)).

Regarding the socioeconomic data collected, women were classified according to age (≤19 years: adolescents; 20 to 34 years: average age; and ≥35 years: advanced age) [25], level of education (<4 years; ≥4 years of study) [26], monthly family income (<1 minimum wage; ≥1 minimum wage), self-declaration of black race (yes or no), and occupation (at home or work outside the home).

The assessment of maternal nutritional status was carried out using height and current weight, measured with the aid of a digital scale with a stadiometer to calculate BMI, and classified according to Atalah Samur et al. [27].

After delivery, information on the newborns was also obtained from the medical records and the declaration of live births, such as weight and length at birth, gestational age at delivery, type of delivery, the Apgar score in the 1st and 5th minutes of life, sex, head circumference (HC), and chest circumference (CC).

The characterization of the newborns was made based on the weight and length at birth and classified following the method of Villar et al. [28] in percentiles, with those below the 10th percentile considered small for gestational age (SGA), those between the 10th and 90th percentiles considered suitable for gestational age (SUGA), and those above the 90th percentile considered large for gestational age (LGA). The classification of the length at birth followed the same pattern as the weight. Additionally, birth weight was also classified according to the criteria proposed by the World Health Organization (WHO) [29], such as low birth weight (LBW) (<2,500 kg), adequate birth weight (≥2,500 kg-<4,000 kg), and macrosomia (≥4,000 kg). To obtain information on the newborn's nutritional status, the CC/HC ratio was calculated and was considered adequate when the value was equal to 1 [30]. In addition, the Apgar score in the 1st and 5th min of life, when the score is higher than 7, is indicative of the child's vitality at birth [31].

### 2.5. Umbilical Cord Samples

The samples were obtained from the central region of the umbilical cord immediately after delivery or, at most, within 20 min, to perform analyses to quantify the biomarkers of redox imbalance and inflammation. After carrying out the appropriate washing procedures, the tissues were stored in a biofreezer at -80°C.

### 2.6. Umbilical Cord Extract Preparation

**Figure 1:**
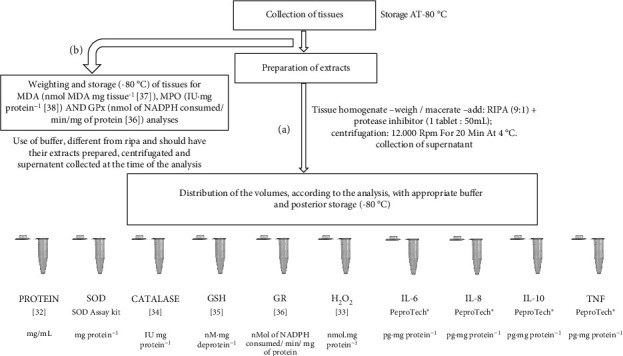
Flowchart representing the steps of collection, preparation and analyses of umbilical cords. Legend: CAT—catalase; GPx—glutathione peroxidase; GR—glutathione reductase; GSH—reduced glutathione; H_2_O_2_—hydrogen peroxide; IL—interleukin; MDA—malondialdehyde; MPO—myeloperoxidase; SOD—superoxide dismutase; TNF—tumor necrosis factor.

Extracts of the umbilical cords were prepared using approximately 100 mg of the tissue. Liquid nitrogen was used to promote tissue disintegration, which facilitates maceration. After this step, the product obtained was transferred to microtubes, and RIPA buffer was added in a volume equal to nine times the weight of the tissue. RIPA buffer (radioimmunoprecipitation assay) contains 50 mM Trizma base, 150 mM NaCl (sodium chloride), and 1 mM ethylenediamine tetraacetic acid (EDTA), as well as the detergents Triton X-100 1%, deoxycholate 1%, and sodium dodecyl sulfate (SDS) 0.1%, with protease inhibitors at pH 7.4 (protease inhibitor cocktail tablets were purchased from Sigma-Aldrich). Subsequently, the homogenates were centrifuged at 12,000 rpm for 12 min at 4°C, and the supernatant was collected as the umbilical cord extracts, which were stored in appropriate volumes for subsequent analyses, in a biofreezer at -80°C. This procedure was used for most analyses, except for MDA, MPO, and GPx (Figure 1).

### 2.7. Protein Quantification

After preparing the extracts, the proteins from the umbilical cord were quantified according to Bradford [32], aiming at normalizing the results of the subsequent analyses by the protein content. For this, 20 *μ*L of the sample, using bovine serum albumin ((BSA) 1 to 5 mg dL^−1^) as the standard, was pipetted into a microplate in duplicate, followed by the addition of 200 *μ*L of the Bradford reagent. After 5 min in the dark, the reading was performed on a spectrophotometer at 595 nm.

### 2.8. Redox Imbalance Biomarkers

SOD was analysed using the SOD Sigma Kit-WST®, following the manufacturer's instructions, with the spectrophotometer reading performed at 450 nm and the activity expressed in mg protein^–1^. Hydrogen peroxide (H_2_O_2_) was analysed according to the method of Pick and Keisari [33], which is based on the ability of H_2_O_2_ to oxidize phenol red, a reaction mediated by radish peroxidase, and the concentration was expressed in nmol mg of protein^−1^. Buffer (phosphate buffer, dextrose, and NaCl, in a proportion of 1 : 25, at pH 7.0) was added to the umbilical cord extracts just before the analyses. Then, 5 *μ*L of phenol red and 4.25 *μ*L of radish peroxidase were added and incubated for 30 min at 37°C. Finally, the samples were transferred to microplates. Then, 25 *μ*L of NaOH was added, and the spectrophotometer reading at 610 nm was obtained. All assays were performed in duplicate.

Catalase (CAT) was evaluated according to Paton et al. [34], as previously described by Aebi [35]. The method consists of monitoring the decomposition rate of H_2_O_2_ in a spectrophotometer at 240 nm, with readings taken every 15 seconds for 5 min, with the results expressed in U of CAT mg of protein^−1^. A CAT unit is defined as the amount of enzyme needed to decompose, at 37°C, 1 *μ*mol min^−1^ of H_2_O_2_. Reduced glutathione (GSH), adapted from Tipple and Rogers [36], uses assay buffer (PBS 0.1 M + 5 mM EDTA, at pH 7.4 and 5% metaphosphoric acid), and the reading is carried out in a spectrophotometer at 412 nm, in 3 min kinetics, with readings every 30 seconds and the results expressed in nM mg of protein^−1^.

Glutathione reductase (GR) and glutathione peroxidase (GPx) were analysed according to a protocol adapted from Flohé and Gunzler [37], with GR activity being directly measured by nicotinamide dinucleotide phosphate (NADPH) as a cofactor in the reduction of GSSG (oxidized glutathione) to GSH. For this analysis, umbilical cord extracts and a freshly prepared reaction medium (0.1 M phosphate buffer (pH 7.0) and 1.0 mM EDTA, GSSG, and NADPH) were used. The analyses were performed in duplicate, and the decrease in absorbance at 340 nm was monitored for 10 min, kinetically, with 10 readings taken every 15 seconds. The results are expressed in nmol of NADPH consumed/min/mg of protein.

For the analysis of GPx, considering its ability to convert H_2_O_2_ into H_2_O and O_2_, *tert*-butyl hydroperoxide (*t*-BuOOH) was used once its dismutation was performed by GPx, generating 2 GSH molecules through the action of GR based on the oxidation of NADPH. Therefore, this assay assesses the consumption of NADPH. The sample preparation was different from that previously described. In summary, approximately 50 mg of the tissue in assay buffer (0.1 M phosphate buffer with 5 mM EDTA, at pH 7.4) was used. The homogenate was centrifuged at 12,000 rpm for 20 min at 4°C, and the supernatant was collected. GR, GSH, *t*-BuOOH, and NADPH were added at the time of analysis. The experiment was performed in a microplate in duplicate, with incubation at 37°C for 10 min, with subsequent spectrometric monitoring of the absorbance decay at 340 nm per min for 5 min, and the result is expressed in nmol of NADPH consumed/min/mg of protein.

Malondialdehyde (MDA) was evaluated by HPLC, measuring the peak height, following the technique of Vickie et al. [38]. The conditions of the HPLC system were a C18 column, a 259 mm length, and a 4.6 mm internal diameter, with a mobile phase of acetonitrile and Trizma buffer (pH 7.4, in a proportion of 1 : 9). The umbilical cord tissue was homogenized in Trizma, *tert*-butyl-hydroxytoluene (BHT), and acetonitrile buffer. Afterward, these homogenates were centrifuged at 3500 rpm for 10 min at 4°C, and the supernatant was later filtered through a specific HPLC filter (0.22 *μ*m). The flow rate was 1.0 mL min^−1^, and the MDA level was calculated using a standard curve, which was generated using 1,1,3,3-tetramethoxypropane (TMP), which is a precursor to MDA, and subsequently corrected for the weight of the tissue analysed (in mg), as shown in the following:

(1) $$\begin{array}{c} \text{Tissue MDA}=\text{MDA found}\times \frac{1000}{\text{tissue weight }\left(\text{mg}\right)}. \end{array}$$

The results are expressed in nmol MDA mg tissue^−1^. The retention time was approximately 2 min and 48 seconds, and the UV detector was adjusted to 270 nm.

### 2.9. Inflammatory Biomarkers

The inflammatory biomarkers interleukin- (IL-) 6, IL-8, IL-10, and tumor necrosis factor-alpha (TNF-*α*), with duplicate analyses, were evaluated by means of an enzyme-linked immunosorbent assay (ELISA) with a PeproTech® kit (PeproTech Brasil FUNPEC, Ribeirão Preto, SP, BR), following the manufacturer's instructions, with cytokine levels expressed in pg mg protein^−1^.

Myeloperoxidase (MPO) activity was measured via adaptation of the method proposed by Bradley et al. [39]. For the analysis, approximately 25 mg of tissue in assay buffer (50 mM potassium phosphate buffer, 0.5% hexadecyltrimethylammonium bromide, and 5 mM EDTA (pH 6.0)) was used. This homogenate was centrifuged at 4,000 rpm for 15 min at 4°C. Then, the supernatant was removed and centrifuged again at 12,000 rpm for 15 min, at 4°C. Afterward, in duplicate, 50 *μ*L of the supernatant was transferred to a microplate, and 50 *μ*L of *ortho*-dianisidine solution (0.68 mg/mL) was added. The incubation was carried out at 37°C for 15 min, and then 50 *μ*L of H_2_O_2_ solution (0.3%) was added. After incubation at the same temperature for 10 min, a spectrophotometer reading was performed at 460 nm. It should be noted that an MPO unit is defined as the amount of H_2_O_2_ decomposed per min. The results are expressed in U of MPO mg protein^-1.^

### 2.10. Statistical Analyses

Statistical analyses were performed using Stata version 13.0 software and IBM Statistical Package for the Social Sciences (SPSS) software 20.0 (SPSS Inc., USA), adopting *α* = 0.05. To compare the socioeconomic, obstetric, and nutritional status characteristics of the studied groups, a chi-square test was performed. To evaluate normality, the Lilliefors test was used. Then, a visual graphical analysis was performed with a QQ plot, and it was decided to use nonparametric investigations due to violations of normality. After this, Wilcoxon–Mann–Whitney tests were performed. Finally, the results of the biomarkers evaluated in this study were related to the perinatal variables (birth weight, gestational age, the Apgar scores in the 1st and 5th min, HC, CC/HC, length at birth, and birth complications) through the multinomial and Poisson regression, adjusting for maternal age, origin, education, family income, gestational BMI, black race, primigravida, mode of delivery, and gestational age. In addition, in order to investigate if there were significant interactions between the different redox/inflammatory markers and PE for each of the outcomes, the interaction term biomarkers∗PE was also included in each outcome regression model, considering *p* < 0.05 as significant.

## 3. Results

### 3.1. Sample Size

In this study, 100 pregnant women with PE (PE group) and 50 pregnant women without the disease were included. As this study is part of a larger study, the achieved sample power was calculated with the G Power program, considering a determination coefficient (*R*^2^) of 0.027 and a sample of 100, with an alpha of 5%, which results in an achieved power of 50.3% in the present study.

### 3.2. PE and without PE Group Characterization

The mean age of the PE group was 25.5 ± 7.04 years; for the group of women without the disease, it was 24.2 ± 6.53 years (*p* = 0.259).  Table 1 summarizes the socioeconomic, obstetric, and nutritional status data of the pregnant women with PE and women without the disease. It is possible to observe that in the PE group, 26% were teenagers, and 13% were older; 8.3% declared themselves to be black; concerning education, 3% had <4 years of study; 24.2% stated that they lived monthly on less than 1 minimum wage per family; in terms of nutritional status, 26.9% and 32.2% were overweight and obese, respectively, and half of them were in their first pregnancy. About folic acid supplementation, 7.8% started before pregnancy and 63.3% started in the first trimester; ferrous supplementation was received by 84.8%. There were no statistically significant differences in these variables between the PE and without PE groups.

Besides, in the PE group, magnesium sulfate supplementation, as an eclampsia-preventive medicine, was used in only 5% of the cases; 78% did not receive this supplementation. About medications, 49% of them used standard hospital medication to control the disease, especially antihypertensive drugs, such as methyldopa and hydralazine.

### 3.3. Newborn Characterization

Regarding the newborn characterization data from PE pregnancies, 52.5% were female, the predominant mode of delivery was cesarean delivery (70.4%), 22.4% of the neonates were premature, 75.3% had an adequate length at birth, and 8.6% and 1.1% had low vitality on the Apgar score in the 1st and 5th min, respectively. Regarding birth weight, 11.5% were classified as SGA and 13.5% as LGA, and the CC/HC ratio was inadequate in 73% of cases (Table 2).

It is important to mention that Tables 1 and 2 are reproduced from a previously published part of the overall study and are repeated here for the reader's convenience. Permission to duplicate them was obtained.

### 3.4. Redox Imbalance and Inflammation Biomarker Levels

**Figure 2:**
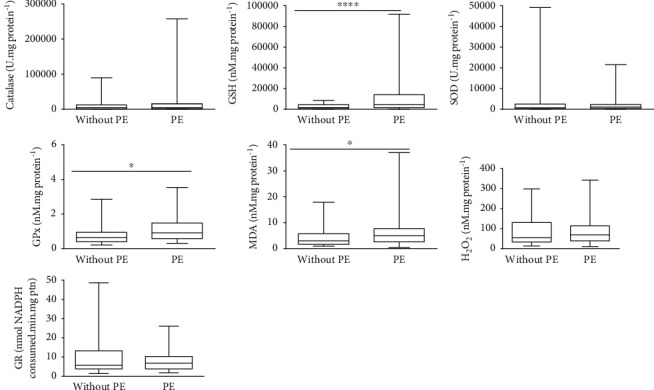
Whisker charts of the biomarkers of redox imbalance between pregnant women with PE and without PE. Legend: CAT—catalase; GPx—glutathione peroxidase; GR—glutathione reductase; GSH—reduced glutathione; H_2_O_2_—hydrogen peroxide; MDA—malondialdehyde; SOD—superoxide dismutase. Mann–Whitney test: *p* < 0.05.

**Figure 3:**
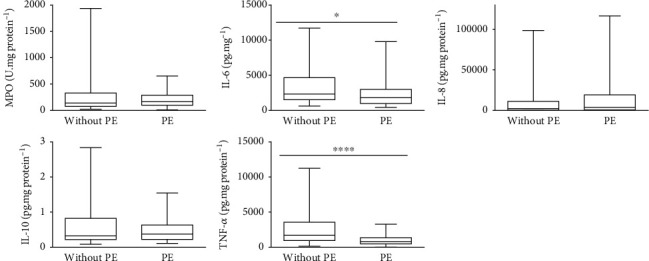
Whisker charts of the biomarkers of inflammation between pregnant women with PE and without PE. Legend: IL—interleukin; MPO—myeloperoxidase; TNF—tumor necrosis factor. Mann–Whitney test: *p* < 0.05.

Figures 2 and 3 show the whiskers charts of redox imbalance and inflammation biomarkers, respectively, between PE and the without PE pregnancies. The umbilical cords of the PE group showed higher levels of GSH (*p* ≤ 0.001), GPx (*p* = 0.016), and MDA (*p* = 0.028), and lower levels of IL-6 (*p* = 0.019), and TNF-*α* (*p* ≤ 0.001), than those of the women without PE group. The other results showed no statistically significant differences.

### 3.5. Association between Redox Imbalance and Inflammation Biomarkers and Perinatal Variables

Tables 3–6 show the results of the associations between the biomarkers of redox imbalance and inflammation in the umbilical cord of PE and without PE pregnancies, and the perinatal variables (birth weight, gestational age, the Apgar scores in the 1st and 5th min, HC, CC/HC, length at birth, and birth complications), with significant associations identified.

In the PE group, the associations identified by perinatal variables were LBW with TNF-*α* and MDA; LGA with TNF-*α*; preterm with TNF-*α* and MDA; a low Apgar score in the 5th min with TNF-*α*, SOD, H_2_O_2_, CAT, GR, GPx, IL-8, and MPO; CC/HC with TNF-*α*, SOD, H_2_O_2_, and GR; a low Apgar score in the 1st min with MDA; macrosomia with SOD; low HC with GR and MPO; and birth complications with GSH.

In turn, the group of women without PE shows the following associations: preterm with SOD, CAT, GR, GPx, IL-6, IL-10, and MPO; a low Apgar score in the 1st min with SOD, GR, IL-10, and MDA; low HC with SOD, CAT, GR, GPx, IL-6, IL-10, and MPO; short length with SOD, CAT, GR, GPx, IL-6, IL-8, IL-10, MPO, MDA, GSH, and H_2_O_2_; birth complications with SOD, GR, GPx, IL-8, MPO, MDA, and GSH; a low Apgar score in the 5th min with CAT, GR, GPx, IL-6, IL-10, and MDA; and CC/HC with CAT and MPO.

Interaction analysis was also performed between the redox imbalance/inflammatory biomarkers and PE (Tables 3–6) for each of the investigated outcomes, where a significant interaction was only seen between the MDA biomarker and LGA newborns (*p* = 0.022) (Table 3, column 11); however, MDA showed a nonsignificant role in the PE group (PR: 0.21; CI: 0.04-1.07) and in the group without PE (PR: 1.02; CI: 0.81-1.27).

## 4. Discussion

Few studies [40–42] in the literature have evaluated biomarkers of oxidative stress and inflammation in the umbilical cord tissues from pregnancies with PE. To our knowledge, the present work is the only one that evaluated a wide variety of such markers, including antioxidants of an enzymatic and nonenzymatic nature and markers of oxidative damage, and analysed their relationship with perinatal outcomes. Higher levels of the antioxidants GSH and GPx and the oxidative tissue damage marker MDA, as well as lower levels of IL-6 and TNF-*α*, were observed in the umbilical cords of PE pregnancies.

It is understood that in PE, there is an imbalance between the enzymatic and low-molecular weight antioxidants (superoxide dismutase (SOD), GPx, catalase; biothiols, and others) and prooxidants (with exacerbation of these), based on the inadequacy of the remodeling of the spiral arteries [9, 43]. Given this scenario, placental hypoxia resulting from impaired trophoblastic invasion raises the Th1 immune response characterized by the production of gamma interferon (IFN-*γ*), TNF-*α*, and IL-2, when compared to Th2 activity, which is characterized by the production of IL-4, IL-5, IL-6, IL-10, and IL-13, in addition to an increase in the Th17 profile, which secretes the proinflammatory cytokine IL-17 and stimulates the migration of other cytokines that act in cellular communication, and oxidative stress, with the production of ROS and RNS [44].

Therefore, it is important to highlight that in PE, there is an increase in oxidative stress and inflammation in a two-way path, where the hypoxia/reperfusion process during inadequate placentation, which leads to inadequate remodeling of the uterine spiral arteries, maintaining its caliber and high resistance, is responsible for initiating the higher production of ROS and proinflammatory cytokines. It is also known that, in the inflammatory response, there is the involvement of genes related to the increase of oxidative stress, especially by the release of the nuclear factor kappa B (NF-*κ*B), located in the cellular cytoplasm. ROS are able to oxidize the I*κ*B kinase (IKK) complex, leading to the delivery of NF-*κ*B, which is formed by p50 and p65 subunits, and so this factor is able to enter the cell nucleus, promoting the transcription of several proinflammatory cytokines such as IL-6 and TNF-*α*. This process is exacerbated in PE, but occurs naturally during gestation. Furthermore, in PE, there is an overactivation of NF-*κ*B, contributing to induce the expression of proinflammatory and antiangiogenic proteins, further elevating oxidative stress, inflammation, and vascular dysfunction [9, 45, 46].

Besides, during the trophoblastic invasion, the decidua contains a lot of necessary immune system cells to promote this process, such as macrophages, natural killer (NK) cells, T cells, and regulatory T cells (Treg). In PE, a higher secretion of proinflammatory cytokines and a decrease of Treg cells occur as a result of an immunological imbalance, leading to the activation of a chronic inflammatory response in the immune system [9, 47, 48].

Among the components of the nonenzymatic oxidative defense system, GSH has a prominent role. It is the major intracellular antioxidant compound, found in abundance in the cytosol, nucleus, and mitochondria, able to perform biotransformation and elimination of xenobiotics and in defense of cells against oxidative stress, which is very important in PE pathology. It exerts antioxidant action through its oxidation to oxidized glutathione (GSSG), in a reaction catalyzed by GPx. In addition, GSH has important effects on various organs of the human body; it plays a variety of roles in detoxification, redox regulation, and cellular signaling; including biological development, fertilization, implantation, and cellular differentiation [8, 9, 12, 49, 50].

Regarding the placenta, the total GSH and GPx levels are higher in the decidua, although they are increased throughout the organ and reflect the maternal protective capacity of this tissue against toxins and free radicals produced by the maternal-foetal interface or by the placenta in relation to the foetus. Both in healthy pregnant women and in PE ones, where there is an increase in redox imbalance, the protective effect of this antioxidant can be observed, including on foetal outcomes [12, 51]. However, the studies are still controversial. It was also reported in the literature that there may be a reduction in the placental levels of GSH in PE in response to the increase in oxidative stress [52].

The findings of the present study revealed, in comparison with the women without PE, higher levels of GSH in the umbilical cord of PE pregnancies. This result suggests that higher production of this antioxidant may be related to an organic compensatory mechanism, aimed at mitigating the consequences of oxidative stress. The presence of oxidative stress in the umbilical cord during PE was revealed by the increased level of MDA in comparison to the group without PE. GSH plays a beneficial role in the health of the foetus, as demonstrated previously in the placenta, where higher levels of GSH in PE placentas positively influenced birth weight, HC, CC, and gestational age at birth, which might be a compensation mechanism against oxidative stress [12]. However, data on this topic are still controversial. Research with blood and placenta that has evaluated GSH levels has produced conflicting results, with some authors observing higher levels of this antioxidant in PE [51, 53] and others finding reduced levels [8, 52, 54–56].

In addition to catalyzing the conversion of GSH to GSSG, the enzyme GPx also helps transforming H_2_O_2_ into water [57]. The findings of our study revealed higher levels of GPx in the umbilical cords of PE pregnancies than in women without PE. This may explain the similar levels of H_2_O_2_ in comparison to normal pregnancies. Therefore, it is possible that the effects of GPx prevented the formation of hydroxyl radicals from H_2_O_2_; thus, the damage to the membrane, revealed by the increase in MDA levels in the cords of women with PE, may be due to other reactive species.

Ferreira et al. [12], after evaluating biomarkers of oxidative stress in placental PE pregnancies compared to normal pregnancies, reported higher levels of SOD and CAT and a higher GSH/GSSG ratio, suggesting the existence of a compensatory mechanism against oxidative stress and a positive relationship of GSH with perinatal outcomes at birth, indicating an important role of this antioxidant in the health of the foetus.

These findings are partly in line with those of the present study, suggesting that the increase in the levels of this antioxidant in PE umbilical cords has occurred to compensate for the oxidative stress present during PE in order to protect the foetus.

Despite the scarcity of data evaluating oxidative stress markers in umbilical cords from PE pregnancies, a study conducted in Turkey assessed MDA levels in the placenta and umbilical cords in women with PE and their relationships to perinatal outcomes, and higher MDA levels were found in both tissues, in addition to a positive correlation between MDA levels in the umbilical cord and the neonate's asphyxia criteria [40]. Thus, the data in the present study are similar to the previous findings, except for the relationship between MDA levels and perinatal outcomes.

It is known that during pregnancy, PE causes oxidative stress, which simultaneously leads to an increase in the inflammatory response mediated by proinflammatory cytokines, especially TNF-*α* and IL-6, and activation of the inflammatory process also leads to more significant oxidative stress [9, 58]. Notably, as a consequence of oxidative stress, there is a release into the maternal circulation of a high amount of syncytial material, antiangiogenic factors, and debris, stimulating the activation of systemic leukocytes, which finally leads to a generalized inflammatory response [59]. Given the above, it is interesting to note that the scientific community widely reports that the exacerbated inflammatory response in PE observed in the blood and in the placenta is an essential determinant of the disease's pathophysiology. However, in this research, lower levels of IL-6 and TNF-*α* were observed in umbilical cords from pregnancies with PE.

TNF-*α* is a cytokine with an inflammatory capacity that activates macrophages, regulates the production of other inflammatory cytokines, and increases the production of lipid mediators [60, 61]. IL-6, in general, is responsible for communicating with monocytes, endothelial cells, and fibroblasts [47, 62]. In healthy pregnancies, the IL-6 and TNF-*α* levels are reduced in placentas and in maternal blood, umbilical cord blood, and the amniotic fluid. As pregnancy progresses and labour begins, their concentrations increase considerably, indicating that the presence of these cytokines in gestational fluids is a maturational event, as they increase with gestational age [63].

In PE, it is proposed that inadequate placentation leads to placental hypoxia, which leads to the expression of higher amounts of proinflammatory cytokines, especially IL-6 and TNF-*α*, which subsequently favours higher production of ROS, in addition to the release of autoantibodies against the angiotensin II type 1 receptor (AT1-AA) and other antiangiogenic factors, such as sFlt-1, soluble endoglin, and endothelin-1. Therefore, these changes induce endothelial dysfunction and clinically characterize PE. Additionally, the increase in placental levels of these interleukins promotes the excessive activation of macrophages and prevents the recruitment of deciduous natural killer cells, which are essential for the remodeling of the spiral arteries, and they stimulate the activity of matrix metalloproteinases, which further inhibit the trophoblast invasion process by degrading the decidual extracellular matrix [64].

There is little information on the levels of inflammatory markers in umbilical cords in PE. Valencia-Ortega et al. [65] reported higher umbilical serum concentrations of IL-6 and TNF-*α* in PE than in the control group. Reyes-Aguilar et al. [66], studying the transcription of proinflammatory cytokine genes in umbilical cord blood, identified lower levels of IL-6 transcripts in PE women than in normotensive women. Thus, the authors suggested that the decrease in the release of inflammatory cytokines by umbilical vein endothelial cells in women with PE is an attempt to protect the foetus. This is clinically significant because neonates of mothers with PE have reduced immune activity, a fact supported by their augmented susceptibility to infections [66].

However, a study carried out in Portugal found a correlation between inflammatory markers in the maternal circulation and umbilical cord blood during PE, including IL-6. Therefore, the increase in maternal inflammation during PE is reflected in higher inflammation in the foetal circulation, which leads to early stimulation of the immune system to combat the increase in circulating cytokines. Lower leukocyte and neutrophil levels have also been observed in mothers' umbilical cord blood with PE, which was interpreted by the authors as a physiological response to try to neutralize the inflammatory process [67]. Thus, the present study's findings suggest that the lower levels of IL-6 and TNF-*α* observed in PE umbilical cords may be the result of an adaptive mechanism to protect the foetus.

The literature is scarce regarding the study of umbilical cord tissues. Therefore, no data related to the specific role of cytokines in this organ could be obtained. However, studies carried out on the placenta emphasize that cytokines play a physiological role in that organ, including modulation of the invasion and differentiation of trophoblasts, placental growth, and metabolic and endocrine homeostasis. In addition, the release of cytokines from placental tissues to the umbilical circulation results in increased concentrations of cytokines in umbilical cord blood and, consequently, in the foetus. It is noteworthy that physiological situations, such as labour, and pathological situations involving hypoxia, as in the case of PE, both lead to greater production of placental cytokines, and both can be reflected in the cord and foetal blood [68–70].

The present study allowed observing some significant associations between biomarkers of redox imbalance and inflammation in umbilical cords and perinatal outcomes. Regarding the biomarkers of redox imbalance in the PE group, a positive association was observed between SOD and a low Apgar score in the 5th min, CAT and a low Apgar score in the 5th min, GR with low HC, and MDA with LBW and preterm birth. A negative association was seen between SOD, macrosomia, and CC/HC ratio < 1; GR with a low Apgar score in the 5^th^ min and CC/HC ratio < 1; GPx with a low Apgar score in the 5th min; GSH with complications at birth; MDA with a low Apgar score in the 1st min of life; and H_2_O_2_ with a low Apgar score in the 5th min and CC/HC ratio < 1. Therefore, it is possible to note that about enzymatic and nonenzymatic antioxidants, the higher the levels of these, the greater protection over perinatal outcomes, including birth weight and CC/HC ratio < 1, which indicates better nutritional status at birth. On the other hand, the cell damage marker MDA showed a relationship with worse health conditions at birth, such as LBW and prematurity.

Concerning inflammation biomarkers, an inverse association was seen between TNF-*α* and LBW, preterm birth and a low Apgar score in the 5th min, and MPO with a low Apgar score in the 5th min. On the other hand, the higher the TNF-*α* level, the greater the occurrence of LGA newborns. IL-8 was directly associated with a low Apgar score in the 5th min. Corroborating with these findings, a direct association was seen between MPO and low HC. Thus, a relationship is observed between higher levels of proinflammatory biomarkers with adverse perinatal outcomes, including a low Apgar score in the 5th min and low HC, reinforcing the existence of an inadequate health condition at birth and insufficient foetal development in PE. Therefore, high levels of proinflammatory mediators are related to adverse perinatal outcomes in PE. As such, once inflammatory biomarkers can negatively influence the proper development of the conceptus and the pregnancy, the inflammation in PE requires an early control and should be the therapeutic target in the clinical monitoring of these pregnant women.

Corroborating with these findings, in a recent study carried out by our group in PE placentas from the same population herein studied, a positive (beneficial) association was observed between placental GSH levels and birth weight, HC, CC, and gestational age at birth [12]. In other words, the higher the placental GHS levels, the better the perinatal outcomes of conceptuses from pregnancies with PE. Such results are considered to reflect a maternal physiological adaptive process, aimed at allowing adequate passage of nutrients through the cord to the foetus, and in this way, favouring an adequate foetal development. The literature has shown that changes in the umbilical cord structure may be associated with pathological conditions and adverse perinatal outcomes, such as gestational diabetes mellitus, PE, IUGR, and foetal death, and that these changes are related to the umbilical cord length and thickness, WJ area, cord insertion type, cord knot, morphometry, and flow parameters of the umbilical vessels [5–7].

With regard to perinatal outcomes, a study evaluating preterm newborns from a university hospital reported that maternal PE generates an increase in neonatal morbidities in preterm infants without causing significant changes in the levels of cytokines in the umbilical blood, given that the levels of cytokines can be altered by other conditions, causing prematurity [11]. In another study, with pregnant women in Turkey, the authors reported higher maternal and umbilical serum levels of IL-6, IL-8, and TNF-*α* in PE, in addition to significantly higher levels of IL-8 and TNF-*α* in newborns with IUGR from PE mothers relative to those who, even with PE, had infants with normal foetal growth [71]. In another cohort study carried out with pregnant women in prenatal care, they observed an increase in the levels of TNF-*α* in the umbilical blood of newborns of mothers diagnosed with PE, although no differences were observed in the maternal serum levels of this cytokine in the first and second trimesters [72]. In turn, a study produced in a high-risk hospital in Turkey identified a serum elevation of IL-6 and TNF-*α* in women with PE and a correlation of these markers with birth weight [73].

Regarding the associations between redox imbalance biomarkers and adverse perinatal outcomes in the group of women without PE, it was seen that the enzymes and antioxidant compounds analysed showed an inverse association with unfavorable health conditions at birth and foetal development, such as prematurity, a low Apgar score in the 1st and 5th min of life, short length, low HC, HC/CC ratio < 1, and complications during delivery, showing the antioxidant protective role of these compounds to foetal health. On the other hand, the higher the levels of the damage marker MDA, the lower the Apgar score in the 1st and 5th min, and the greater the occurrence of short length at birth, indicating the possibility of the involvement of this biomarker with impaired foetal development and health conditions at birth.

The relationship between inflammation biomarkers and perinatal outcomes assessed in the group of women without PE indicated the protective role of IL-10 against prematurity, a low Apgar score in the 1st and 5th min of life, low HC, and a short length. On the other hand, increased MPO levels were associated with complications at birth. In turn, the IL-6, IL-8, and TNF-*α* levels were associated with better perinatal outcomes at birth, including gestational age, weight, length, HC at birth, the Apgar score, and even the complications during childbirth, emphasizing the occurrence of better foetal conditions at birth in women without PE.

After performing the interaction analysis between the PE group and the group without PE, a significant interaction was seen between the MDA biomarker and LGA newborns, as shown in Table 3. So, there was only one significant interaction, but it was an outcome that did not show significance with the groups alone. Thus, although the MDA is higher in the umbilical cords of PE pregnancies, this is not enough to justify an action of this marker that is different from the action it normally exerts in pregnant women without the disease.

In view of the above and comparing the findings of the present study with the previous study carried out by our group on placentas of PE women [12], it is possible to observe that the reduced levels of IL-6 in the umbilical cord of pregnancies with PE, as well as the increase of this cytokine in placentas, indicate the accumulation of IL-6 in the placenta for foetal protection. On the other hand, TNF-*α* was reduced in the umbilical cord of PE pregnancies; however, in placentas, there were no differences between the PE and without PE groups. With this, we hypothesized that the cord tissue is without protection against inflammation in PE, and more tissues must be researched in this condition since there was low inflammation in the evaluated tissues, and proinflammatory cytokines may have been recruited to another organ.

Given the above, it is possible to identify some limitations in the present study, which include nonassessment of food consumption and the lack of measurement of markers of endothelial damage, such as sFlt-1, soluble endoglin, and endothelin-1, which have been suggested by the literature as possibly involved in PE pathophysiology. We also report that the sample size power calculated at 50.3% is considered low, which can also be a study limitation. Still, we were able to find significant results in some of the analysis, indicating that the low power of the primary analysis was not able to compromise the other analysis in our study.

Despite the appointed limitations, the present study suggests a potential compensation mechanism that protects the newborn of a mother with PE from the stress process experienced in pregnancy, which seems to have an evolutionary advantage in protecting the foetus exposed to oxidative damage during labour, as evidenced in the placenta [12]. In an additional study, not directly related to the placenta/umbilical cord but indirectly related to the health of the foetus, Silberstein et al. [74] reported a significant decrease (approximately 20%) in lipid peroxidation levels (MDA) in the colostrum of women who had PE compared with the control group and an increase in polyphenol concentrations (approximately 33%), an important antioxidant, highlighting a possible compensatory effect where the body activates the defense system, which can be a physiological organic adaptation to prioritize and protect the child.

As such, the results found in this research showed an increase in the levels of GSH, GPx, and MDA, in addition to a reduction in the levels of IL-6 and TNF-*α* in PE cords compared to women without PE. They can serve as subsidies to guide clinical management of PE, as well as for future research on pathophysiology and perinatal outcomes resulting from the disease. Furthermore, the importance of the umbilical cord against the oxidative damage and inflammation present in PE is evident, providing protection of the health of the foetus, although an increase in the oxidative damage marker MDA was noted. Therefore, the existence of a compensatory mechanism developed by the umbilical cord is suggested to protect the health of the foetus during PE.

Future perspectives from the present study include a comparison of the present data on PE pregnancy, using similar biomarkers obtained from other biological matrixes, such as urine, saliva, and blood, which may facilitate the clinical routine, once they can be assessed throughout pregnancy. Additionally, the use of the umbilical cord may be considered together with the placenta, in these studies, once they give a real picture of the mother/foetal health at the moment of birth.

## 5. Conclusion

Higher levels of GSH and GPx, in addition to lower levels of IL-6 and TNF-*α*, in the PE umbilical cord may result from an adaptive mechanism to maintain the oxidative and inflammatory balance; however, despite these changes, damage to the cell membrane occurred since the MDA content was higher. Besides, it is clear that this redox imbalance does not directly influence the outcome of the pregnancy, confirming that other physiological and adaptive mechanisms may act to preserve foetal health. Therefore, it is suggested that the umbilical cord plays an important role in controlling redox imbalance and inflammation in pregnancies with PE. The present results also reinforce the necessity for continuous research on GSH as a protective compound for the perinatal outcome, favouring a possible supplementation to increase GSH levels, especially in PE women. It will also stimulate research on umbilical cords.

## Figures and Tables

**Table 1:** Socioeconomic, obstetric, and nutritional status characteristics of preeclampsia pregnant women and women without preeclampsia in Maceió, Alagoas, Brazil, in 2017.

	Preeclampsia		Without preeclampsia		OR	CI 95%
	*n*	%	*n*	%		
	100	66.7	50	33.3		
*Age (years)*						
≤19	26	26.0	16	32.0	0.747	0.355-1.570
20-35	61	61.0	31	62.0	1.084	0.542-2.168
≥35	13	13.0	3	6.0	2.341	0.635-8.629
*Self-declaration of black race*						
Yes	8	8.3	7	14.0	0.534	0.182-1.568
No	88	91.7	43	86.0		
No information	4		0			
*Education (years)*						
<4	3	3.0	2	4.0	0.742	0.120-4.529
≥4	97	97.0	48	96.0		
*Monthly family income (minimum wage)*						
<1	23	24.2	12	26.7	0.878	0.391-1.976
≥1	72	75.8	33	73.3		
No information	5		5			
*Primiparous*						
Yes	50	50.0	21	42.0	1.381	0.696-2.739
No	50	50.0	29	58.0		
*Previous PE*						
Yes	23	23.0	1	2.7	10.753	1.397-82.769
No	77	77.0	36	97.3		
*Gestational BMI*						
Low weight	6	6.5	8	17.4	0.345	0.112-1.060
Eutrophy	32	34.4	17	37.0	0.957	0.461-1.985
Overweight	25	26.9	12	26.1	1.103	0.497-2.450
Obesity	30	32.2	9	19.5	2.063	0.886-5.989
No information	7		4			
*Folic acid supplementation before pregnancy*						
Yes	7	7.8	4	8.9	0.86	0.23-3.12
No	83	92.2	41	91.1		
No information	10		5			
*Folic acid supplementation in the first trimester*						
Yes	57	63.3	31	70.5	0.72	0.33-1.57
No	33	36.7	13	29.5		
No information	10		6			
*Ferrous sulfate supplementation*						
Yes	78	84.8	40	85.1	0.97	0.36-2.60
No	14	15.2	7	14.9		
No information	8		3			

Legend: BMI—body mass index; PE—preeclampsia; CI—confidence interval; OR—odds ratio. Chi-square test, *p* < 0.05. Source: data on preeclampsia was reported by Ferreira et al. [12], with permission.

**Table 2:** Characteristics of newborns from pregnancies with preeclampsia in Maceió, Alagoas, Brazil, in 2017.

Variables	PE	
	*n* = 100	
	*n*	%
*Sex*		
Men	52	52.5
Women	47	47.5
No information	1	
*Mode of delivery*		
Cesarean	69	70.4
Normal	29	29.6
No information	2	
*Gestational age at birth*		
Preterm	22	22.4
Term	76	77.6
Postterm	0	0.0
No information	2	
*Birth weight*		
SGA	11	11.5
SUGA	72	75.0
LGA	13	13.5
No information	4	
*Length at birth*		
Low	8	9.0
Adequate	67	75.3
High	14	15.7
No information	11	
*Apgar 1st minute*		
≤6	8	8.6
≥7	85	91.4
No information	7	
*Apgar 5th minute*		
≤6	1	1.1
≥7	92	98.9
No information	7	
*CC/HC ratio*		
Adequate	24	27.0
Inadequate	65	73.0
No information	11	

Legend: CC—chest circumference; HC—head circumference; LGA—large for gestational age; PE—preeclampsia; SGA—small for gestational age; SUGA—suitable for gestational age. Source: data reported in Ferreira et al. [12], with permission.

**(a):** 

Redox imbalance and inflammatory markers	Birth weight									
	Small for gestational age					Large for gestational age				
	PE		Without PE		*p*-for-interaction	PE		Without PE		*p*-for-interaction
	OR	CI	OR	CI		OR	CI	OR	CI	
SOD	0.99	0.99-1.00	1.00	0.01-81.32	0.483	0.99	0.99-1.00	0.99	0.99-1.00	0.216
H_2_O_2_	0.99	0.97-1.00	1.02	0	0.771	1.00	0.99-1.01	1.00	0.99-1.02	0.998
CAT	0.99	0.99-1.00	1.00	0.25-3.97	0.411	0.99	0.99-1.00	1.00	0.99-1.00	0.389
GSH	1.00	0.99-1.00	1.00	0.14-6.72	0.382	0.99	0.99-1.00	0.99	0.99-1.00	0.665
GR	0.99	0.85-1.14	0.95	0	0.971	1.05	0.97-1.14	1.00	0.82-1.22	0.913
GPx	3.11	0.60-16.03	8.87	0	0.241	1.33	0.49-3.59	1.03	0.21-4.92	0.897
MDA	1.13	0.91-1.42	0.79	0	0.144	0.21	0.04-1.07	1.02	0.81-1.27	**0.022**
IL-6	1.00	0.99-1.00	1.00	7.25*e*‐10–1.38*e* + 09	0.469	1.00	0.99-1.00	1.00	0.99-1.00	0.679
IL-8	0.99	0.99-1.00	0.99	0.14-6.68	0.290	0.99	0.99-1.00	0.99	0.99-1.00	0.167
IL-10	0.73	0.06-8.74	10.86	0	0.987	2.27	0.54-9.53	2.44	0.17-35.19	0.833
TNF-*α*	0.99	0.99-1.00	1.00	5.22*e*‐07–1918116	0.639	1.00	**1.00-1.00**	1.00	0.99-1.00	0.875
MPO	0.99	0.99-1.00	1.00	0.00-60179.64	0.565	1.00	0.99-1.00	0.99	0.99-1.00	0.361

**(b):** 

Redox imbalance and inflammatory markers	Birth weight									
	Low birth weight					Macrosomia				
	PE		Without PE		*p*-for-interaction	PE		Without PE		*p*-for-interaction
	OR	CI	OR	CI		OR	CI	OR	CI	
SOD	0.99	0.99-1.00	0.99	0.99-1.00	0.954	0.99	**0.99-0.99**	—	—	0.994
H_2_O_2_	0.99	0.98-1.00	1.02	0.99-1.05	0.819	1.00	0.98-1.01	—	—	1.000
CAT	0.99	0.99-1.00	0.98	0	0.522	0.99	0.99-1.00	—	—	1.000
GSH	1.00	0.99-1.00	0.99	0.99-1.00	0.138	0.99	0.99-1.00	—	—	0.999
GR	0.96	0.87-1.05	9.54*e*‐13	0	0.347	0.97	0.83-1.13	—	—	1.000
GPx	0.96	0.42-2.17	1.9*e*‐164	0	0.306	0.22	0.02-1.87	—	—	1.000
MDA	1.23	**1.02-1.50**	0.62	0.26-1.48	0.068	0.58	0.17-1.92	—	—	0.999
IL-6	0.99	0.99-1.00	0.99	0.99-1.00	0.264	0.99	0.99-1.00	—	—	1.000
IL-8	0.99	0.99-1.00	0.98	0	0.482	0.99	0.99-1.00	—	—	0.999
IL-10	0.50	0.10-2.50	0	0	0.282	0.64	0.04-9.58	—	—	1.000
TNF-*α*	0.99	**0.99-0.99**	0.66	0	0.196	1.00	0.99-1.00	—	—	1.000
MPO	0.99	0.99-1.00	0.95	0.89-1.01	0.358	1.00	0.99-1.00	—	—	1.000

Legend: CAT—catalase; CI—confidence interval; GPx—glutathione peroxidase; GR—glutathione reductase; GSH—reduced glutathione; H_2_O_2_—hydrogen peroxide; IL—interleukin; MDA—malondialdehyde; MPO—myeloperoxidase; OR—odds ratio; SOD—superoxide dismutase; TNF—tumor necrosis factor. Multinomial regression: *p* < 0.05. Adjusted for maternal age, origin, education, family income, gestational BMI, black race, primigravida, way of delivery, and gestational age. *Note*. *p*-for-interaction: *p* value for the interaction term between the redox imbalance/inflammatory marker and PE, for each of the investigated outcomes.

**Table 4:** Association between biomarkers of redox imbalance and inflammation in umbilical cords and perinatal outcomes such as preterm birth and the low Apgar scores in the 1st min and 5th min of pregnancies with and without preeclampsia at a university hospital in Maceió, Alagoas, Brazil, in 2017.

Redox imbalance and inflammatory markers	Outcomes														
	Preterm birth					Low Apgar score 1st (min)					Low Apgar score 5th (min)				
	PE		Without PE		*p*-for-interaction	PE		Without PE		*p*-for-interaction	PE		Without PE		*p*-for-interaction
	PR	CI	PR	CI		PR	CI	PR	CI		PR	CI	PR	CI	
SOD	0.99	0.99-1.00	**0.99**	**0.99-0.00**	0.756	**0.99**	0.99-1.00	**1.00**	**1.00-1.00**	0.675	**1.00**	**1.00-1.00**	**0.99**	0.99-1.00	—
H_2_O_2_	**1.00**	0.99-1.00	**1.00**	0.99-.00	0.363	**1.00**	0.99-1.00	**0.99**	0.97-1.01	0.352	**0.97**	**0.95-0.98**	**0.98**	0.95-1.00	—
CAT	**0.99**	0.99-1.00	**0.99**	**0.99-0.99**	0.203	**0.99**	0.99-1.00	**1.00**	0.99-1.00	0.842	**1.00**	**1.00-1.00**	**0.99**	**0.99-0.99**	—
GSH	1.00	0.99-1.00	0.99	0.99-1.00	0.393	**0.99**	0.99-1.00	**0.99**	0.99-1.00	0.416	**—**	**—**	**0.99**	0.99-1.00	—
GR	**1.00**	0.95-1.05	**0.88**	**0.81-0.97**	0.414	**1.02**	0.95-1.10	**0.14**	**0.11-0.19**	0.236	**0.76**	**0.76-0.76**	**0.34**	**0.11-0.19**	—
GPx	**1.19**	0.77-1.82	**0.36**	**0.17-0.75**	0.582	**1.09**	0.39-3.05	**0.00**	**0.00-0.00**	0.221	**0.25**	**0.07-0.79**	**0.00**	**0.00-0.00**	—
MDA	**1.15**	**1.07-1.23**	**0.91**	0.81-1.03	0.181	**0.81**	0.68-0.96	**0.56**	**0.52-0.62**	0.451	**0.80**	**0.61-1.05**	**0.43**	**0.43-0.43**	—
IL-6	0.99	0.99-1.00	**0.99**	**0.99-0.99**	0.124	1.00	0.99-1.00	0.99	0.99-1.00	0.151	0.99	0.99-1.00	**0.99**	**0.99-0.99**	—
IL-8	1.00	0.99-1.00	0.99	0.99-1.00	0.366	**1.00**	0.99-1.00	**1.00**	0.99-1.00	0.538	**1.00**	**1.00-1.00**	**0.99**	0.99-1.00	—
IL-10	**1.04**	0.39-2.76	**0.09**	**0.018-0.515**	0.450	**1.49**	0.38-5.77	4.36**e**‐07	**5.08-0.00**	0.215	**0.05**	0.00-1.80	4.34**e**‐09	**1.09-1.72**	—
TNF-*α*	**0.99**	**0.99-0.99**	**0.99**	**0.99-0.99**	0.482	**1.00**	0.99-1.00	**0.99**	0.98-0.99	0.209	**0.99**	**0.99-0.99**	**0.99**	0.98-0.99	0.988
MPO	1.00	0.99-1.00	**0.99**	**0.99-0.99**	0.694	**1.00**	0.99-1.00	**1.00**	0.99-1.00	0.157	**0.99**	**0.99-0.99**	**1.00**	0.99-1.00	—

Legend: CAT—catalase; CC/HC—ratio chest circumference and head circumference; CI—confidence interval; GPx—glutathione peroxidase; GR—glutathione reductase; GSH—reduced glutathione; HC—head circumference; H_2_O_2_—hydrogen peroxide; IL—interleukin; MDA—malondialdehyde; MPO—myeloperoxidase; PR—prevalence ratio; SOD—superoxide dismutase; TNF—tumor necrosis factor. Poisson regression: *p* < 0.05. Adjusted for maternal age, origin, education, family income, gestational BMI, black race, primigravida, way of delivery, and gestational age. *Note*. *p*-for-interaction: *p* value for the interaction term between the redox imbalance/inflammatory marker and PE, for each of the investigated outcomes.

**Table 5:** Association between biomarkers of redox imbalance and inflammation in umbilical cords and perinatal outcomes such as low head circumference, CC/HC ratio < 1, and low length at birth of pregnancies with and without preeclampsia at a university hospital in Maceió, Alagoas, Brazil, in 2017.

Redox imbalance and inflammatory markers	Outcomes														
	Low head circumference					CC/HC < 1					Low length at birth				
	PE		Without PE		*p*-for-interaction	PE		Without PE		*p*-for-interaction	PE		Without PE		*p*-for-interaction
	PR	CI	PR	CI		PR	CI	PR	CI		PR	CI	PR	CI	
SOD	0.99	0.99-1.00	**0.99**	**0.99-0.99**	0.085	**0.99**	**0.99-0.99**	**0.99**	0.99-1.00	0.456	0.99	0.99-1.00	**0.99**	**0.99-0.99**	0.652
H_2_O_2_	**1.00**	0.99-1.00	**0.95**	**0.93-0.98**	0.315	**0.99**	**0.99-0.99**	**1.00**	0.99-1.00	0.092	**0.99**	0.97-1.00	**0.37**	**0.35-0.39**	0.379
CAT	**0.99**	0.99-1.00	**0.99**	**0.99-0.99**	0.208	**0.99**	0.99-1.00	**0.99**	**0.99-0.99**	0.357	0.99	0.99-1.00	**0.99**	**0.99-0.99**	0.447
GSH	**0.99**	0.99-1.00	**0.99**	0.99-1.00	0.221	1.00	0.99-1.00	0.99	0.99-1.00	0.882	1.00	0.99-1.00	**0.99**	**0.99-0.99**	0.730
GR	**1.04**	**1.00-1.08**	2.9**e**‐176	8.8**e**‐216–9.6**e**137	0.087	**0.96**	**0.94-0.99**	**1.00**	0.97-1.03	0.395	**0.99**	0.86-1.14	**0.68**	**0.55-0.85**	0.834
GPx	1.01	0.47-2.19	1.70**e**‐48	**—**	0.184	**1.02**	0.78-1.34	**1.06**	0.86-1.30	0.759	**1.23**	0.48-3.17	**0.23**	**0.07-0.74**	0.543
MDA	**1.01**	0.90-1.12	**0.94**	0.79-1.11	0.264	**1.00**	0.96-1.04	**1.01**	0.99-1.03	0.503	**1.04**	0.96-1.13	**1853.957**	**957.6-3589.0**	0.465
IL-6	0.99	0.99-1.00	**0.99**	**0.98-0.99**	0.251	0.99	0.99-1.00	1.00	0.99-1.00	0.324	0.99	0.99-1.00	**0.99**	**0.99-0.99**	0.620
IL-8	**0.99**	0.99-1.00	**0.99**	**0.99-0.99**	0.395	0.99	0.99-1.00	1.00	0.99-1.00	0.522	0.99	0.99-1.00	**1.00**	**1.00-1.00**	0.220
IL-10	**2.22**	0.95-5.13	2.7**e**‐179	1.8**e**‐194–4.2**e**164	0.106	**0.62**	0.39-1.00	**1.24**	0.85-1.81	0.143	**0.98**	0.08-11.64	**0.01**	**0.00-0.20**	0.835
TNF-*α*	**1.00**	0.99-1.00	**0.99**	**0.99-0.99**	0.188	**0.99**	**0.99-0.99**	**1.00**	0.99-1.00	0.163	**0.99**	0.99-1.00	**0.99**	**0.99-0.99**	0.604
MPO	**1.00**	**1.00-1.00**	**0.96**	**0.93-0.98**	0.126	**0.99**	0.99-1.00	**0.99**	**0.99-0.99**	0.114	**1.00**	0.99-1.00	**0.95**	**0.91-0.99**	0.834

Legend: CAT—catalase; CC/HC—ratio chest circumference and head circumference; CI—confidence interval; GPx—glutathione peroxidase; GR—glutathione reductase; GSH—reduced glutathione; HC—head circumference; H_2_O_2_—hydrogen peroxide; IL—interleukin; MDA—malondialdehyde; MPO—myeloperoxidase; PR—prevalence ratio; SOD—superoxide dismutase; TNF—tumor necrosis factor. Poisson regression: *p* < 0.05. Adjusted for maternal age, origin, education, family income, gestational BMI, black race, primigravida, way of delivery, and gestational age. *Note*. *p*-for-interaction: *p* value for the interaction term between the redox imbalance/inflammatory marker and PE, for each of the investigated outcomes.

**Table 6:** Association between biomarkers of redox imbalance and inflammation in umbilical cords and perinatal outcome birth complications of pregnancies with and without preeclampsia at a university hospital in Maceió, Alagoas, Brazil, in 2017.

Redox imbalance and inflammatory markers	Outcome				
	Birth complications				
	PE		Without PE		*p*-for-interaction
	PR	CI	PR	CI	
SOD	**0.99**	0.99-1.00	**1.00**	**1.00-1.00**	0.260
H_2_O_2_	**1.00**	0.99-1.01	**0.98**	0.94-1.01	0.579
CAT	0.99	0.99-1.00	1.00	0.99-1.00	0.935
GSH	**0.99**	**0.99-1.00**	**0.99**	**0.99-0.99**	0.318
GR	**1.03**	0.95-1.11	**0.44**	**0.35-0.55**	0.259
GPx	**0.76**	0.18-3.16	**0.00**	**4.45-0.00**	0.278
MDA	**1.12**	0.84-1.51	**0.31**	**0.22-0.43**	0.870
IL-6	1.00	0.99-1.00	0.99	0.99-1.00	0.195
IL-8	0.99	0.99-1.00	**0.99**	**0.99-0.99**	0.223
IL-10	**1.62**	0.39-6.65	**0.00**	**—**	0.177
TNF-*α*	1.00	0.99-1.00	0.99	0.99-1.00	0.324
MPO	**1.00**	0.99-1.00	**1.00**	**1.00-1.00**	0.216

Legend: CAT—catalase; CC/HC—ratio chest circumference and head circumference; CI—confidence interval; GPx—glutathione peroxidase; GR—glutathione reductase; GSH—reduced glutathione; HC—head circumference; H_2_O_2_—hydrogen peroxide; IL—interleukin; MDA—malondialdehyde; MPO—myeloperoxidase; PR—prevalence ratio; SOD—superoxide dismutase; TNF—tumor necrosis factor. Poisson regression. *p* < 0.05. Adjusted for maternal age, origin, education, family income, gestational BMI, black race, primigravida, way of delivery, and gestational age. *Note*. *p*-for-interaction: *p* value for the interaction term between the redox imbalance/inflammatory marker and PE, for each of the investigated outcomes.

## Data Availability

Data are available on request through Prof. Alane Cabral Menezes de Oliveira, Universidade Federal de Alagoas, Faculdade de Nutrição. E-mail: alanecabral@gmail.com. Phone: +55 (82) 999766895. Fax: +55 (11) 55739525.
